# Unexpected frequency of the pathogenic *AR* CAG repeat expansion in the general population

**DOI:** 10.1093/brain/awad050

**Published:** 2023-02-17

**Authors:** Matteo Zanovello, Kristina Ibáñez, Anna-Leigh Brown, Prasanth Sivakumar, Alessandro Bombaci, Liana Santos, Joke J F A van Vugt, Giuseppe Narzisi, Ramita Karra, Sonja W Scholz, Jinhui Ding, J Raphael Gibbs, Adriano Chiò, Clifton Dalgard, Ben Weisburd, The American Genome Center (TAGC) consortium, Genomics England Research Consortium, Project MinE ALS Sequencing Consortium, The NYGC ALS Consortium, John C Ambrose, Prabhu Arumugam, Roel Bevers, Marta Bleda, Freya Boardman-Pretty, Christopher R Boustred, Helen Brittain, Mark J Caulfield, Georgia C Chan, Greg Elgar, Tom Fowler, Adam Giess, Angela Hamblin, Shirley Henderson, Tim J P Hubbard, Rob Jackson, Louise J Jones, Dalia Kasperaviciute, Melis Kayikci, Athanasios Kousathanas, Lea Lahnstein, Sarah E A Leigh, Ivonne U S Leong, Javier F Lopez, Fiona Maleady-Crowe, Meriel McEntagart, Federico Minneci, Loukas Moutsianas, Michael Mueller, Nirupa Murugaesu, Anna C Need, Peter O’Donovan, Chris A Odhams, Christine Patch, Mariana Buongermino Pereira, Daniel Perez-Gil, John Pullinger, Tahrima Rahim, Augusto Rendon, Tim Rogers, Kevin Savage, Kushmita Sawant, Richard H Scott, Afshan Siddiq, Alexander Sieghart, Samuel C Smith, Alona Sosinsky, Alexander Stuckey, Mélanie Tanguy, Ana Lisa Taylor Tavares, Ellen R A Thomas, Simon R Thompson, Arianna Tucci, Matthew J Welland, Eleanor Williams, Katarzyna Witkowska, Suzanne M Wood, Wouter Van Rheenen, Sara L Pulit, Annelot M Dekker, Ahmad Al Khleifat, William J Brands, Alfredo Iacoangeli, Kevin P Kenna, Ersen Kavak, Maarten Kooyman, Russell L McLaughlin, Bas Middelkoop, Matthieu Moisse, Raymond D Schellevis, Aleksey Shatunov, William Sproviero, Gijs H P Tazelaar, Rick A A Van der Spek, Perry T C Van Doormaal, Kristel R Van Eijk, Joke Van Vugt, A Nazli Basak, Ian P Blair, Jonathan D Glass, Orla Hardiman, Winston Hide, John E Landers, Jesus S Mora, Karen E Morrison, Stephen Newhouse, Wim Robberecht, Christopher E Shaw, Pamela J Shaw, Philip Van Damme, Michael A Van Es, Naomi R Wray, Ammar Al-Chalabi, Leonard H Van den Berg, Jan H Veldink, Michael G Hanna, Linda Greensmith, Hemali Phatnani, Jan H Veldink, Bryan J Traynor, James Polke, Henry Houlden, Pietro Fratta, Arianna Tucci

**Affiliations:** Department of Neuromuscular Diseases, Queen Square Institute of Neurology, UCL, London WC1N 3BG, UK; William Harvey Research Institute, Barts and The London School of Medicine and Dentistry, Queen Mary University of London, London EC1M 6BQ, UK; Department of Neuromuscular Diseases, Queen Square Institute of Neurology, UCL, London WC1N 3BG, UK; Department of Neuromuscular Diseases, Queen Square Institute of Neurology, UCL, London WC1N 3BG, UK; Department of Neuromuscular Diseases, Queen Square Institute of Neurology, UCL, London WC1N 3BG, UK; ‘Rita Levi Montalcini’ Department of Neuroscience, University of Turin, Turin 10126, Italy; Neurogenetics Unit, National Hospital for Neurology and Neurosurgery, London WC1N 3BG, UK; Department of Neurology, UMC Utrecht Brain Center, University Medical Center Utrecht, Utrecht University, Utrecht 3508, The Netherlands; Center for Genomics of Neurodegenerative Disease, New York Genome Center, New York, NY 10013, USA; Laboratory of Neurogenetics, National Institute on Aging, National Institutes of Health, Bethesda, MD 20892, USA; Department of Neurology, Brain Sciences Institute, Baltimore, MD 21287, USA; Department of Neurology, Brain Sciences Institute, Baltimore, MD 21287, USA; Neurogenetics Branch, National Institute of Neurological Disorders and Stroke, National Institutes of Health, Bethesda, MD 20892, USA; Laboratory of Neurogenetics, National Institute on Aging, National Institutes of Health, Bethesda, MD 20892, USA; Laboratory of Neurogenetics, National Institute on Aging, National Institutes of Health, Bethesda, MD 20892, USA; ‘Rita Levi Montalcini’ Department of Neuroscience, University of Turin, Turin 10126, Italy; Department of Anatomy, Physiology and Genetics, School of Medicine, Uniformed Services University of the Health Sciences, Bethesda, MD 20814, USA; Program in Medical and Population Genetics, Broad Institute of MIT and Harvard, Cambridge, MT 02142, USA; Department of Neuromuscular Diseases, Queen Square Institute of Neurology, UCL, London WC1N 3BG, UK; Department of Neuromuscular Diseases, Queen Square Institute of Neurology, UCL, London WC1N 3BG, UK; Center for Genomics of Neurodegenerative Disease, New York Genome Center, New York, NY 10013, USA; Department of Neurology, UMC Utrecht Brain Center, University Medical Center Utrecht, Utrecht University, Utrecht 3508, The Netherlands; Laboratory of Neurogenetics, National Institute on Aging, National Institutes of Health, Bethesda, MD 20892, USA; Department of Neurology, Brain Sciences Institute, Baltimore, MD 21287, USA; Neurogenetics Unit, National Hospital for Neurology and Neurosurgery, London WC1N 3BG, UK; Department of Neuromuscular Diseases, Queen Square Institute of Neurology, UCL, London WC1N 3BG, UK; Neurogenetics Unit, National Hospital for Neurology and Neurosurgery, London WC1N 3BG, UK; Department of Neuromuscular Diseases, Queen Square Institute of Neurology, UCL, London WC1N 3BG, UK; Department of Neuromuscular Diseases, Queen Square Institute of Neurology, UCL, London WC1N 3BG, UK; William Harvey Research Institute, Barts and The London School of Medicine and Dentistry, Queen Mary University of London, London EC1M 6BQ, UK

**Keywords:** androgen receptor, whole-genome sequencing, bioinformatics, population genetics, spinal and bulbar muscular atrophy

## Abstract

CAG repeat expansions in exon 1 of the *AR* gene on the X chromosome cause spinal and bulbar muscular atrophy, a male-specific progressive neuromuscular disorder associated with a variety of extra-neurological symptoms. The disease has a reported male prevalence of approximately 1:30 000 or less, but the *AR* repeat expansion frequency is unknown. We established a pipeline, which combines the use of the ExpansionHunter tool and visual validation, to detect *AR* CAG expansion on whole-genome sequencing data, benchmarked it to fragment PCR sizing, and applied it to 74 277 unrelated individuals from four large cohorts. Our pipeline showed sensitivity of 100% [95% confidence interval (CI) 90.8–100%], specificity of 99% (95% CI 94.2–99.7%), and a positive predictive value of 97.4% (95% CI 84.4–99.6%). We found the mutation frequency to be 1:3182 (95% CI 1:2309–1:4386, *n* = 117 734) X chromosomes—10 times more frequent than the reported disease prevalence. Modelling using the novel mutation frequency led to estimate disease prevalence of 1:6887 males, more than four times more frequent than the reported disease prevalence. This discrepancy is possibly due to underdiagnosis of this neuromuscular condition, reduced penetrance, and/or pleomorphic clinical manifestations.

## Teaser

CAG repeat expansions in the *AR* gene cause spinal and bulbar muscular atrophy. Zanovello *et al*. establish a pipeline to detect *AR* CAG expansion from whole-genome sequencing data, and find the mutation frequency to be much higher than the reported disease prevalence, possibly due to underdiagnosis or pleomorphic manifestations.

## Introduction

Spinal and bulbar muscular atrophy (SBMA), also known as Kennedy’s disease, occurs when the CAG repeat coding for a polyglutamine tract in exon 1 of the androgen receptor (*AR*) gene expands beyond 37 repeats.^1^ SBMA fully manifests only in males, with a mean age at onset of 43 years, which is partially influenced by CAG repeat size^2^ and is characterized by progressive muscular weakness induced by the degeneration of the lower motor neurons and primary muscular damage.^1^ Importantly, SBMA is also associated with a variety of non-neurological conditions, including insulin resistance, fatty liver disease, and metabolic syndrome.^3^

The information on the frequency of repeat expansion disorders has relied on epidemiology studies or PCR screening of selected populations. Epidemiological studies report a 1:30 303 or less prevalence amongst male populations,^4–6^ but SBMA is often reported to be underdiagnosed. However, an epidemiological study in the Vasa region of Finland reported 13 cases in a population of 85 000 males (1:6538), although this was attributed to a founder effect^7^; two studies based on PCR sizing in selected populations reported an unexpected high frequency of this genetic defect, namely a PCR screening of a European population, which found the mutation frequency to be 1:6888 X chromosomes^8^; and a meta-analysis of 86 datasets based on PCR sizing reported a population frequency of 1:3703.^9^

Although next-generation sequencing and public genomic data repository technologies have allowed the frequency of single nucleotide variants to be estimated precisely across very large populations,^10^ the inability to reliably size short tandem repeats (STRs) from whole-genome sequencing (WGS) has not permitted the same information to be gathered for STR expansions, which are a major cause of neurogenetic disorders including SBMA. Recently developed bioinformatics tools, such as ExpansionHunter, allow the sizing of STRs from WGS data.^11^

Given the unexpected findings from population studies and considering the limitation of PCR sizing and the use of selected populations, we sought to investigate the frequency of the genetic variant underlying SBMA in the general population by exploiting WGS and using clinically curated public genomic data repositories. We validated this approach, applied it to the 100,000 Genomes Project (100k GP) cohort^12^ and replicated it on three other large WGS datasets (Table 1 and Supplementary Table 1).

**Table 1:** *AR* repeat expansion frequency

Cohort	Gender	Phenotype category	Total participants	Total X chromosomes	Total expansions ≥38	X chromosome frequency ≥38 (95% CI)
100k GP	Male	Non–neuro	13 072	13 072	2	1/6536 (1793–23 833)
	Female	All	20 400	40 800	11	1/3709 (2071–6642)
gnomAD	Male	All	14 947	14 947	5	1/2989 (1277–6998)
	Female	All	14 116	28 232	11	1/2567 (1433–4596)
NIH	Male	Ctrl	1529	1529	1	1/1529 (271–8661)
	Female	All	5176	10 352	2	1/5176 (1420–18 874)
MinE	Male	Ctrl	1272	1272	2	1/636 (175–2319)
	Female	All	3765	7530	3	1/2510 (854–7380)
Summary	Male	*–*	30 820	30 820	10	1/3082 (1674–5674)
	Female	*–*	43 457	86 914	27	1/3219 (2213–4683)
	All	*–*	**74 277**	**117 734**	**37**	**1/3182 (2309–4386)**

The summary result is highlighted in bold.

## Materials and methods

### Whole genome sequencing and *AR* genotyping

#### Whole-genome sequencing and cohort characterization

Supplementary Table 1 provides a summary of age and ethnicity of the cohorts assessed in this study. WGS data including chemistry, read length, coverage, alignment, genome build, and ExpansionHunter version from each cohort are summarized in Supplementary Table 2.

#### *AR* genotyping

ExpansionHunter (Illumina Inc., CA, USA) software was used to estimate repeat lengths of the *AR* CAG disease-causing expansions in samples that had undergone WGS. This algorithm has been validated using experimentally-confirmed samples carrying pathogenic expansions.^13,14^ Pathogenic alleles in the *AR* gene were defined as those containing 38 or more CAG repeats.^1^

#### Visual inspection

**Figure 1:**
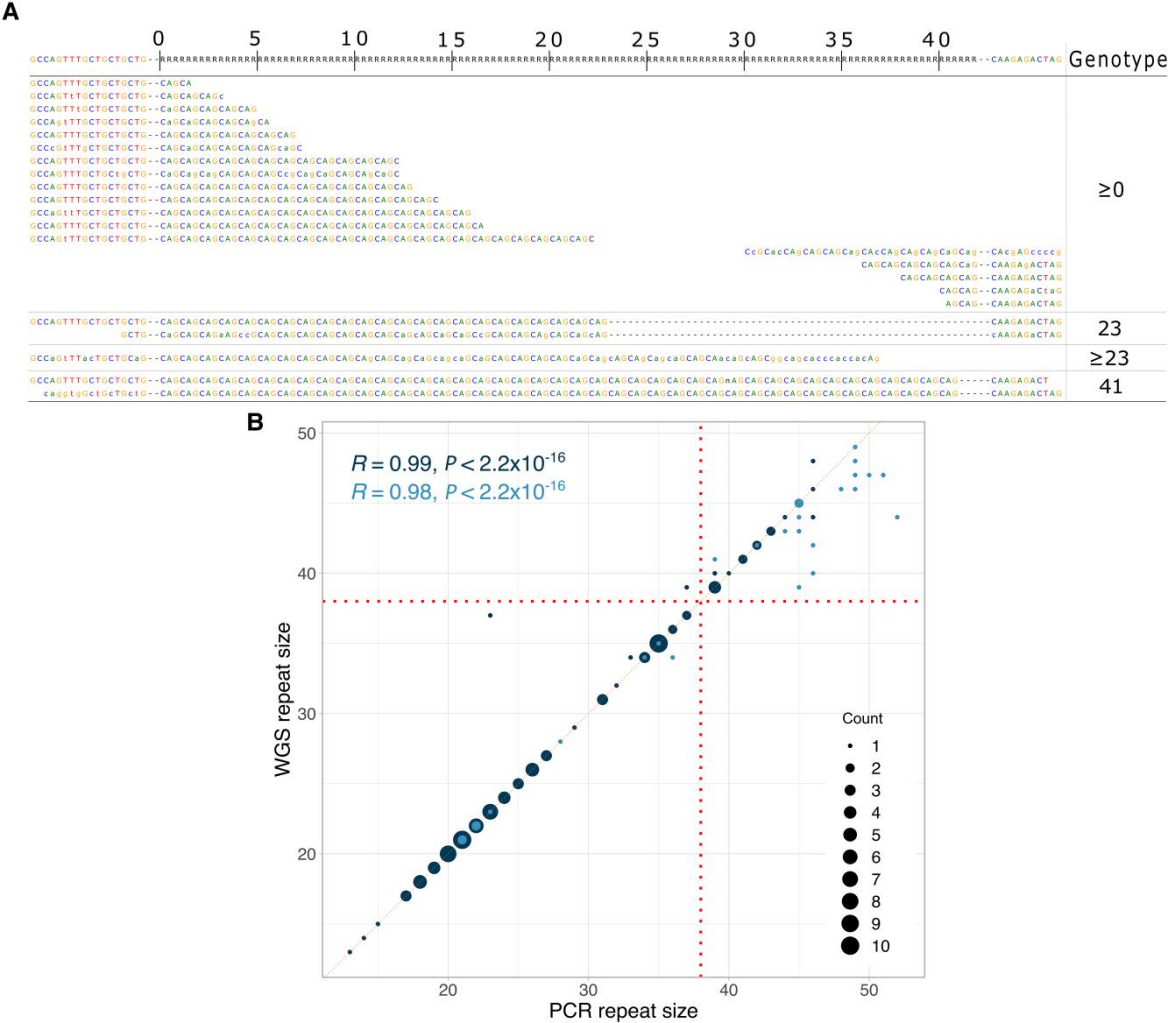
**Validation of the WGS pipeline.** (**A**) Visualization of repeat expansion reads from ExpansionHunter shows reads revealing 23 and 41 CAG repeat alleles. (**B**) Comparison of repeat size estimation between WGS pipeline and PCR. *n* = 133 alleles. Dark points indicate length confirmed by reads spanning the whole repeat and both the flanking sides; light points indicate length confirmed by reads spanning part of the repeat and one flanking side.

As previously validated,^13,15^ Expansion Hunter calls for *AR* CAG repeats underwent a blind quality check process by visual inspection. The ExpansionHunter calls can be visualized by generating ‘pileup’ graphs, which enable the reviewer to easily evaluate the number of reads and the sequences supporting each call, and therefore assess the length of the repeat expansion, as shown in Fig. 1A. A total of 486 pileups were checked, of which there were 282 from 100k GP cohort (≥34 repeats), 67 from NIH (≥34 repeats), 14 from Project MinE (≥37 repeats), and 123 from GnomAD (≥37 repeats). See Supplementary Table 1 for ExpansionHunter calls before and after the visual quality check in each cohort.

### *AR* detection by WGS benchmarking

To assess the performance of WGS to detect the CAG repeat in the *AR* gene, we benchmarked our WGS calls against PCR fragment analysis, obtained as follows.

WGS was obtained from 20 individuals with previously identified pathogenic expansion in *AR* by standard diagnostic PCR testing (i.e. positive control, Supplementary Fig. 1 and Supplementary Table 3, validation ID: NYGC 1–20; 22 alleles from 18 males and two females).

Furthermore, we obtained PCR fragment analysis results for 56 patients recruited to the 100k GP that had been tested previously for the *AR* expansion (i.e. negative controls, Supplementary Fig. 1 and Supplementary Table 3, validation id: GE_1, 2, 3, 4, 5, 6, 7, 8, 9, 10, 11, 12, 13, 14, 15, 16, 17, 18, 19, 20, 21, 22, 23, 24, 25, 26, 27, 30, 31, 32, 33, 35, 36, 37, 38, 39, 43, 45, 46, 47, 48, 51, 52, 54, 55, 59, 61, 63, 64, 66, 67, 69, 72, 73, 74, 75; 79 alleles from 33 males and 23 females).

We also assessed by PCR 21 DNA samples from patients recruited to the 100k GP, where WGS/ExpansionHunter predicted the presence of an expansion (Supplementary Fig. 1 and Supplementary Table 3, validation id: GE_28, 29, 34, 40, 41, 42, 44, 49, 50, 53, 56, 57, 58, 60, 62, 65, 68, 70, 71, 76, 77; 32 alleles from 10 males and 11 females).

### PCR

The CAG trinucleotide repeat length in *AR* was quantified using a PCR method, where *AR* alleles were amplified by PCR using GoTaq DNA polymerase (Promega), with the forward primer (6FAM-GCCTGTTGAACTCTTCTGAGC) containing a fluorescein amidite (FAM)-label, used to enable fluorescence detection during the fragment analysis, and the reverse primer GCTGTGAAGGTTGCTGTTCCTC.^16^ PCR products were electrophoresed on an ABI 3730xl DNA analyser with a LIZ-500 size standard (Applied Biosystems). Fragment analysis was performed with GeneMapper software (version 5.0, Applied Biosystems), deriving numbers of repeats from a standard curve generated using samples of known repeat size ascertained by Sanger sequencing.

### Statistical analysis

The statistical formulas used to assess the repeat expansion performance dataset have been taken from https://www.medcalc.org/calc/diagnostic_test.php. Considering TN = true negative; FP = false positive; TP = true positive; FN = false negative; PPV = positive predictive value:


(1) $$sensitivity=\frac{TP}{TP+FN}$$

(2) $$specificity=\frac{TN}{TN+FP}$$

(3) $$PPV=\frac{sensitivity\;\times \;prevalence}{\left(sensitivity*prevalence)+(1-specificity)\;\times \;(1-prevalence\right)}$$

The R correlation coefficient was calculated using Pearson’s equation:


(4) $$r=\frac{\left(x_{i}-x)(y_{i}-y\right)}{\sqrt{{\left(x_{i}-x\right)}^{2}{\left(y_{i}-y\right)}^{2}}}$$

where *r* = correlation coefficient; *x_i_* = values of the *x*-variable in a sample; *x* = mean of the values of the *x*-variable; *y_i_* = values of the *y*-variable in a sample; *y* = mean of the values of the *y*-variable.

95% CIs for the X chromosome frequencies were computed using the Wilson score method:


(5) $$p=\;\frac{\hat{\;p}+(z_{\alpha /2}^{2}/2n)+z_{\alpha /2}\sqrt{\left((\hat{\;p}(1-\hat{\;p}))/n)+(z_{\alpha /2}^{2}/4n^{2}\right)}}{1+(z_{\alpha /2}^{2}/n)}$$

where *p* = confidence interval for the proportion; $\hat{\;p}$ = estimated proportion; $z_{\alpha /2}$ = statistical test; *n* = cohort numerosity.

### Disease prevalence estimation

**Figure 2:**
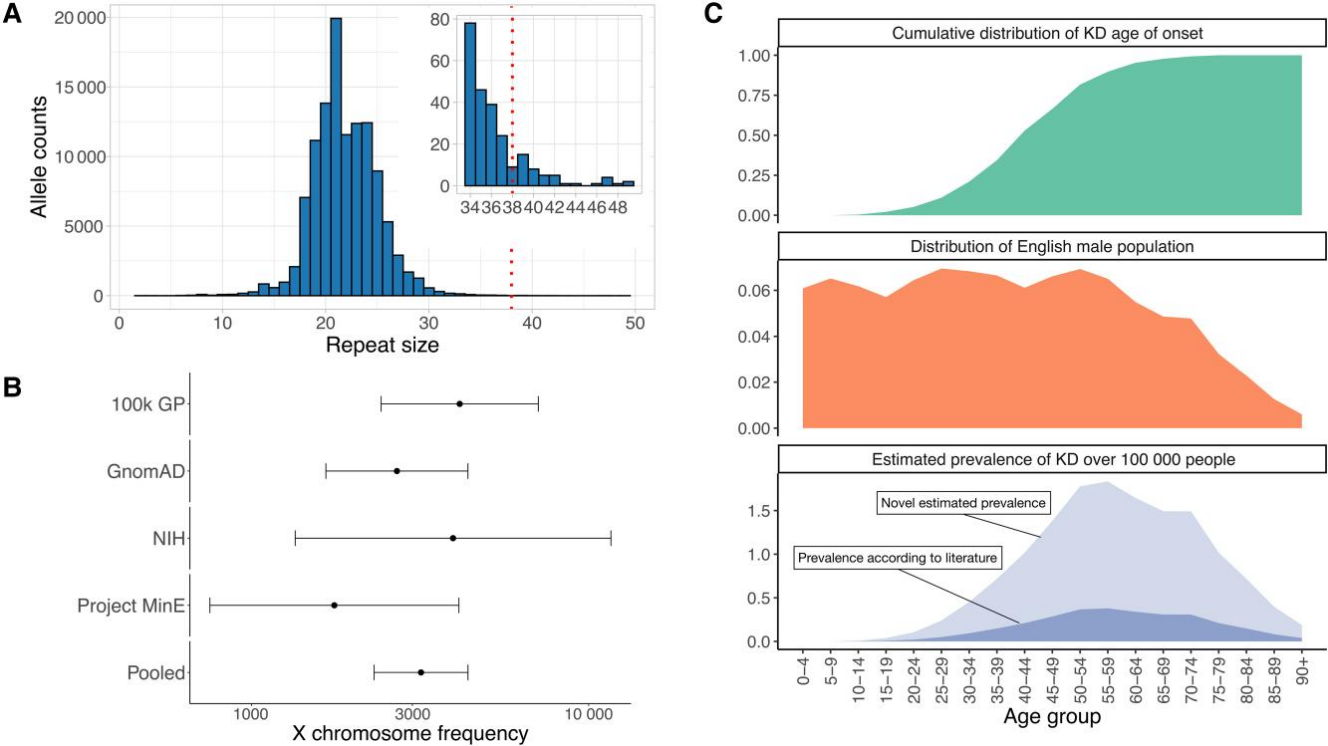
**WGS pipeline detects increased *AR* CAG expansion in four large cohorts.** (**A**) Allele size distribution across 75 035 100k GP genomes; *inset* highlights the distribution of alleles containing ≥34 repeats. (**B**) Frequency estimation of *AR* CAG expansion. WGS pipeline detects 1:3182 *AR* expansion ≥38 repeats in the pooled 100k GP, gnomAD, NIH, and MinE cohorts. Error bars show 95% CI. (**C**) *Top*) Cumulative distribution of SBMA age of onset for *n* = 983 KD cases from the most recent KD meta-analysis; (*middle*) distribution of the English male population (*n* = 27 827 831); and (*bottom*) resulting estimated prevalence of SBMA by age group, considering the literature reported male prevalence of 1:30 303 or less (dark area), and the novel estimated prevalence according to our WGS result (1:6887 males, light area).

We tabulated the cumulative distribution of disease onset reported for 983 patients,^9^ binning them in 5-year age groups (Fig. 2C, top). We also plotted the distribution of the general English male population (*n* = 27 827 831),^17^ using the same 5-year age group bins (Fig. 2C, middle). We then multiplied the cumulative distribution of the disease onset by the corresponding general male count for each age group, to obtain the distribution of the disease by age group, which we then use to estimate the disease prevalence.

### Haplotyping

Starting from the genomic variant call format (gVCF) files from the 100k GP individuals with more than 37 CAG repeats and a European genetic background (*n* = 24, of which 13 males and 11 females), we created merged VCFs for males and females, respectively. We then used gvcfgenotyper to select variants with a sex-adjusted minimum allele frequency (MAF) of 5% within the region comprising 579 kb before and 145 kb after the *AR* CAG repeat (ChrX:66 965 021–67 875 619, GRCh38).^18^ We repeated the process on *n* = 14 346 controls, of which there were 6631 males and 7715 females. Using plink, we created the case input files for Haploview, which were used to shortlist the variants using the tagger function. We then employed the resulting 31 variants to shortlist from a merged VCF file with data from both cases and controls (*n* = 14 370), creating the input files for the formal analysis, performed with Haploview. Within our cohort, we applied the following exclusion criteria: (i) Hardy-Weinberg equilibrium *P*-value for controls <0.001; (ii) genotyping rate >99%; and (iii) MAF >0.01.

### Data availability

Primary data from the 100k GP, which are held in a secure Research Environment, are available to registered users. Please see https://www.genomicsengland.co.uk/about-gecip/for-gecip-members/data-and-data-access for further information.

## Results

### A sensitive and specific pipeline to detect *AR* CAG expansions

Our WGS analysis pipeline to analyse the *AR* expansion combines ExpansionHunter with visual validation of positive results, in accordance with recent guidelines from the American College of Medical Genetics (Fig. 1A).^13,15^

We benchmarked our pipeline against the gold standard diagnostic method, PCR fragment analysis. We used 133 alleles from 97 samples where the WGS pipeline identified PCR-confirmed expanded (*n* = 38) and normal (*n* = 94) alleles, resulting in a sensitivity of 100% (95% CI 90.8–100%), specificity 99% (95% CI 94.2–99.7%), and positive predictive value of 97.4% (95% CI 84.4–99.6%) (Table 2, Supplementary Fig. 1 and Supplementary Table 3).

**Table 2:** Sensitivity, specificity, and positive predictive value for *AR* pathogenic expansion detection

Parameter	Value (95% CI)
Sensitivity	100% (90.8–100%)
Specificity	99.0% (94.2–99.7%)
Positive predictive value	97.4% (84.4–99.6%)

Size estimation correlation yielded *R =* 0.99 (*P* < 2.2 × 10^−16^), with high accuracy in alleles with less than 38 repeats, whilst larger repeats were determined to be in the pathogenic range, but less accurately sized as previously shown (Fig. 1B and Supplementary Fig. 2).^14^

### Unexpected frequency of pathogenic *AR* CAG expansions in the UK population

The 100k GP sequenced the whole genomes of people with a wide range of rare diseases and cancers in the National Health Service in England. Individuals were recruited with their family members where available.^12^ The *AR* allele size distribution in 75 035 individuals from this cohort showed a typical bell shape with a peak at 21 repeats (Fig. 2A and Supplementary Fig. 3).

Analysis of 40 412 unrelated individuals within this cohort identified 25 people carrying pathogenic repeats (≥38 repeats), including 11 females and 14 males. Clinical data available for each individual recruited to the 100k GP, including ICD-10 codes and Human Phenotype Ontology (HPO) terms, were reviewed. Of the 14 males, seven proved to have a clinically confirmed diagnosis of SBMA, whilst all remaining individuals were under 21 years of age, except for one recruited for retinal disorders (Supplementary Table 4). None of the female carriers, who can generally develop mild symptoms, had HPO terms associated with neuromuscular conditions.

To estimate the frequency of *AR* pathogenic expansions, we analysed the repeat size in all unrelated female and male individuals. To avoid overestimating the frequency due to individuals being recruited because of SBMA-related symptoms, we excluded all males recruited under ‘neurological disorders’. We found the X chromosome frequency of the pathogenic expansion to be 1:6536 (95% CI 1:1793–1:23 833, *n* = 13 072) and 1:3709 (95% CI 1:2071–1:6642, *n* = 40 800*)* in males and females respectively (Table 1 and Fig. 2B).

### Multiple large cohorts confirm *AR* CAG expansion frequency

Given the surprisingly high frequency of the *AR* repeat expansion, we sought to carry out our analysis on replication datasets, using North American (NIH and gnomAD) and European (Project MinE) cohorts, where control and neurodegenerative diseases were sequenced with WGS^10,19^ (Supplementary Table 2). The *AR* expansion frequency was 1:2989 and 1:2567 X chromosomes in all males (*n* = 14 947) and all females (*n* = 28 232), respectively, in the gnomAD cohort, 1:1529 and 1:5176 X chromosomes in control males (*n* = 1529) and all females (*n* = 10 352), respectively, in the NIH cohort, and 1:636 and 1:2510 X chromosomes in control males (*n* = 1272) and all females (*n* = 7530), respectively, in the MinE cohort (Fig. 2B and Supplementary Fig. 4). Estimates of *AR* expansion frequency from these cohorts fall within the 95% CI of the frequency estimated in our 100k GP discovery cohort.

A pooled analysis resulted in an overall frequency of 1:3182 X chromosomes (95% CI 1:2309–1:4386, *n* = 117 734) (Table 1 and Supplementary Table 1). Notably, the results with a threshold of 37 repeats, which is known to cause SBMA, were even higher at 1:1899 X chromosomes (95% CI 1:1482–1:2434) (Supplementary Fig. 5 and Supplementary Table 1).

### A discrepancy between expected disease prevalence and current diagnoses

The expected prevalence of the disease is lower than the mutation frequency, as SBMA is an adult-onset disease. We, therefore, used SBMA age of onset distribution^9^ and the general English male population age distribution^17^ with our genetic frequency data to estimate disease prevalence (Fig. 2C). Surprisingly, our results estimated SBMA prevalence at 1:6887 males, more than 4-fold more frequent than previous patient-based epidemiological studies.^4–6^ To rule out a founder effect, as seen in the Finnish study,^7^ we performed a haplotype analysis on European samples from the 100k GP, which resulted in non-significant associations (Supplementary Fig. 6).

## Discussion

Overall, our work identifies an unexpected frequency of the *AR* pathogenic expansion in a UK cohort and confirms this finding using three other large European and North American datasets. Previous findings of an epidemiological study in the Vasa region and a meta-analysis are in line with our findings. Importantly, our use of WGS data allowed us to curate our dataset for relatedness and perform a haplotype analysis that rules out founder effects.

The discrepancy between patient numbers and the frequency of the genetic defect may be due to (i) underdiagnosis of this neuromuscular condition; (ii) variable disease expressivity/reduced penetrance; (iii) pleomorphic clinical manifestations; or (iv) a combination of these factors.

Underdiagnosis of the disease has frequently been suggested, and, whilst the classic disease manifestation with bulbar and limb weakness, highly elevated creatine kinase levels, and gynaecomastia is very typical, the disease can manifest with only certain symptoms and often with a negative family history due to its X-linked mode of transmission, favouring misdiagnosis.^1,7^

Differently from other STR expansion disorders showing incomplete penetrance for all the repeat lengths,^20^ SBMA is reported to be incompletely penetrant between 35 and 37 repeats, but fully penetrant from 38.^1^ Moreover, although strong variability in manifestations and severity of SBMA can occur within siblings, reports of incomplete penetrance within families of SBMA patients are lacking. A recent meta-analysis raised the hypothesis that the *AR* CAG repeat is partially penetrant up to 45 repeats,^9^ although the fact that in the 100k GP all the males older than 45 years, with more than 37 repeats, had an SBMA phenotype argues against reduced penetrance as being the main driver of the discrepancy between patient numbers and mutation frequency. Larger numbers and more targeted studies will be needed to fully clarify this.

Lastly, SBMA has been associated with a number of common non-neurological disorders such as insulin resistance, non-alcoholic fatty liver disease, and metabolic syndrome,^3^ and in light of the frequency of the genetic defect, it should likely be considered in people with these conditions.

In conclusion, we identified an unexpectedly high frequency of the SBMA genetic defect in European and North American populations, suggesting SBMA is underdiagnosed and highlighting how testing may be relevant not only to neuromuscular diseases.

## Supplementary Material

awad050_Supplementary_Data

## Appendix 1

### The Genomics England Research Consortium

John C. Ambrose, Prabhu Arumugam, Roel Bevers, Marta Bleda, Freya Boardman-Pretty, Christopher R. Boustred, Helen Brittain, Mark J. Caulfield, Georgia C. Chan, Greg Elgar, Tom Fowler, Adam Giess, Angela Hamblin, Shirley Henderson, Tim J. P. Hubbard, Rob Jackson, Louise J. Jones, Dalia Kasperaviciute, Melis Kayikci, Athanasios Kousathanas, Lea Lahnstein, Sarah E. A. Leigh, Ivonne U. S. Leong, Javier F. Lopez, Fiona Maleady-Crowe, Meriel McEntagart, Federico Minneci, Loukas Moutsianas, Michael Mueller, Nirupa Murugaesu, Anna C. Need, Peter O’Donovan, Chris A. Odhams, Christine Patch, Mariana Buongermino Pereira, Daniel Perez-Gil, John Pullinger, Tahrima Rahim, Augusto Rendon, Tim Rogers, Kevin Savage, Kushmita Sawant, Richard H. Scott, Afshan Siddiq, Alexander Sieghart, Samuel C. Smith, Alona Sosinsky, Alexander Stuckey, Mélanie Tanguy, Ana Lisa Taylor Tavares, Ellen R. A. Thomas, Simon R. Thompson, Arianna Tucci, Matthew J. Welland, Eleanor Williams, Katarzyna Witkowska, Suzanne M. Wood.

### Project MinE ALS Sequencing Consortium

Wouter Van Rheenen, Sara L. Pulit, Annelot M. Dekker, Ahmad Al Khleifat, William J. Brands, Alfredo Iacoangeli, Kevin P. Kenna, Ersen Kavak, Maarten Kooyman, Russell L. McLaughlin, Bas Middelkoop, Matthieu Moisse, Raymond D. Schellevis, Aleksey Shatunov, William Sproviero, Gijs H. P. Tazelaar, Rick A. A. Van der Spek, Perry T. C. Van Doormaal, Kristel R. Van Eijk, Joke Van Vugt, A. Nazli Basak, Ian P. Blair, Jonathan D. Glass, Orla Hardiman, Winston Hide, John E. Landers, Jesus S. Mora, Karen E. Morrison, Stephen Newhouse, Wim Robberecht, Christopher E. Shaw, Pamela J. Shaw, Philip Van Damme, Michael A. Van Es, Naomi R. Wray, Ammar Al-Chalabi, Leonard H. Van den Berg, Jan H. Veldink.
