# Supplementary material for: Unexpected frequency of the pathogenic AR CAG repeat expansion in the general population
Source: Brain. 2023 Feb 17;146(7):2723–9. doi: 10.1093/brain/awad050 (PMC10316764; doi:10.1093/brain/awad050)

Unexpected frequency of the pathogenic *Androgen Receptor* CAG repeat expansion in the general population

Supplementary tables

Supplementary Table 1: Pipeline calls before and after visual validation, with the threshold set at 38 and 37 repeats, plus demographics

Supplementary Table 2: Sequencing data

Supplementary Table 3: PCR validation results

Supplementary Table 4: Clinical data for 100K GP samples

Supplementary figures

Supplementary Figure 1: Pileups used for validation of the WGS pipeline (ExpansionHunter + visual validation) against PCR. See **Figure 1B** and **eTable 1** for results. N=133 alleles from N=97 samples.

Supplementary Figure 2: Correlation between WGS pipeline and PCR, as for **Figure 1C,** but showing the results for repeat size below **(a)** and above **(b)** 38, according to the WGS pipeline. Dark blue, length confirmed by reads spanning the whole repeat and both the flanking sides; light blue, length confirmed by reads spanning part of the repeat and one flanking side.

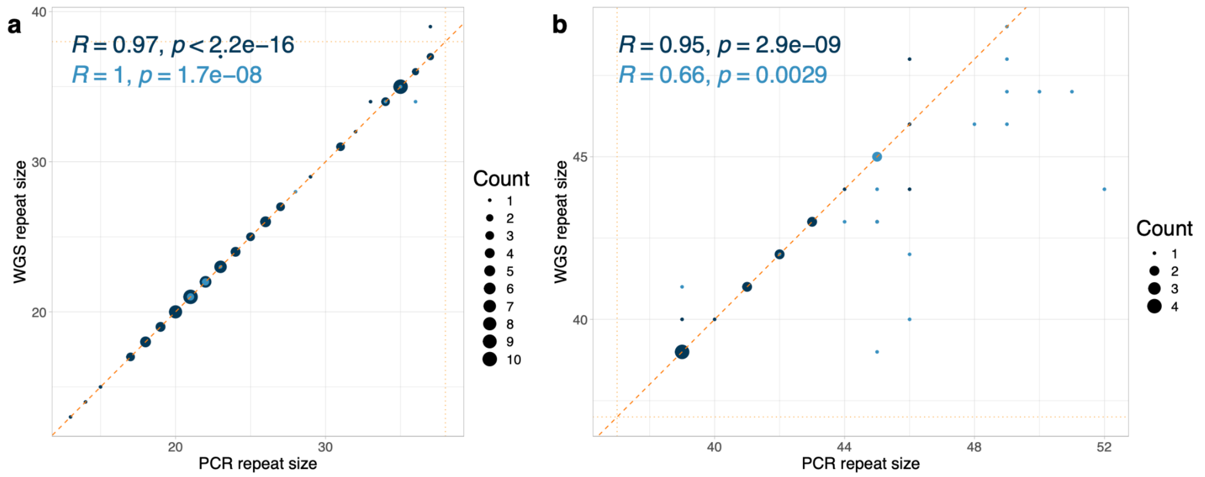

Supplementary Figure 3: Size distribution for **(a)** male alleles, **(b)** female shorter and **(c)** longer alleles, and for **(d)** individuals below 20 years old and **(e)** above 20 years old across 100K GP genomes.

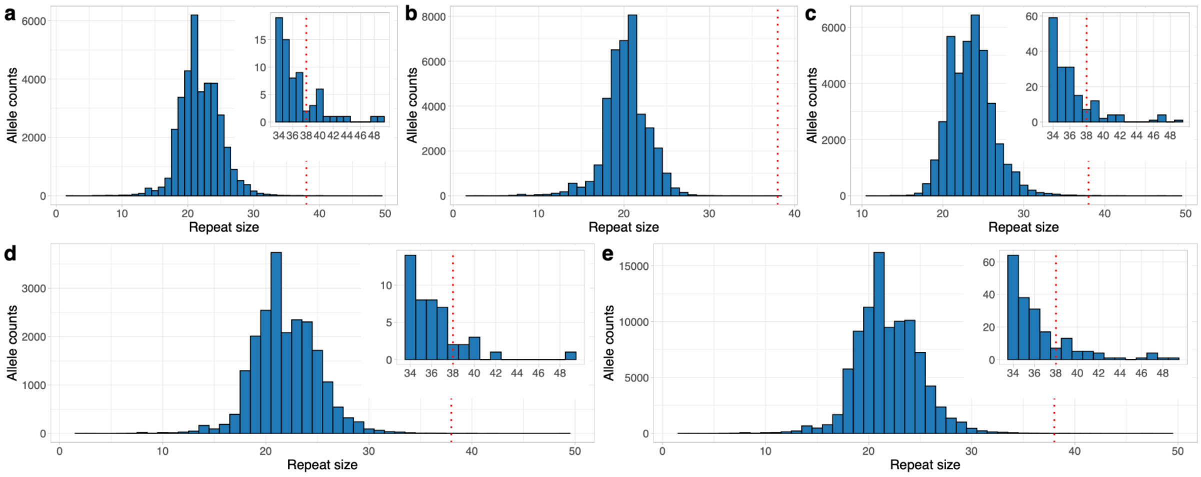

Supplementary Figure 4: Frequency estimation of *AR* CAG expansion, as for **Figure 1E**, detailed with male and female subgroups for each cohort (grey).

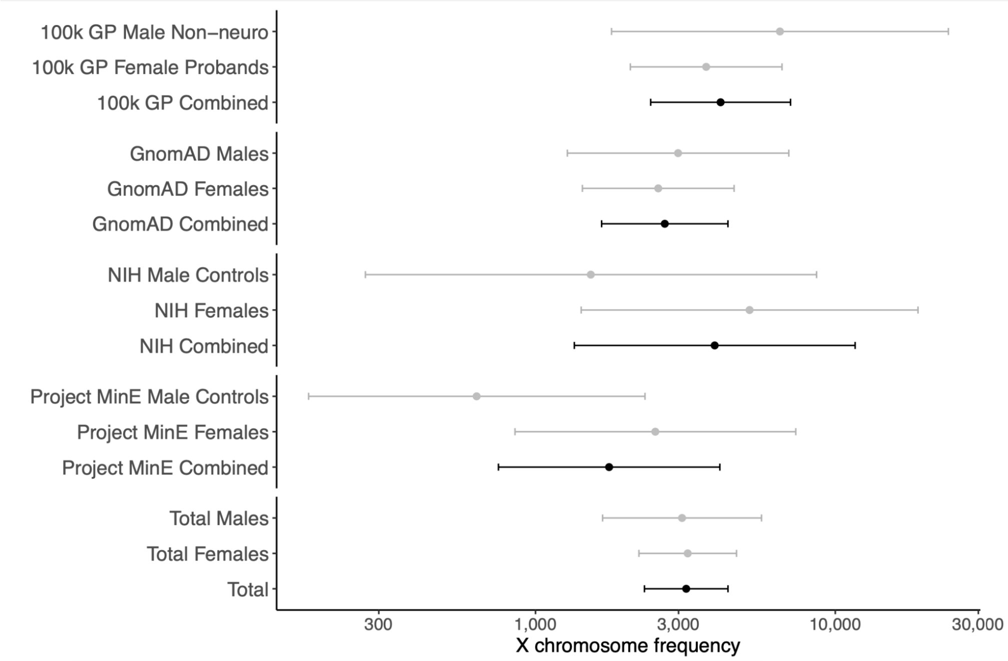

Supplementary Figure 5: Frequency estimation of *AR* CAG expansion, as for **eFigure 4**, but with the threshold set at 37 repeats.

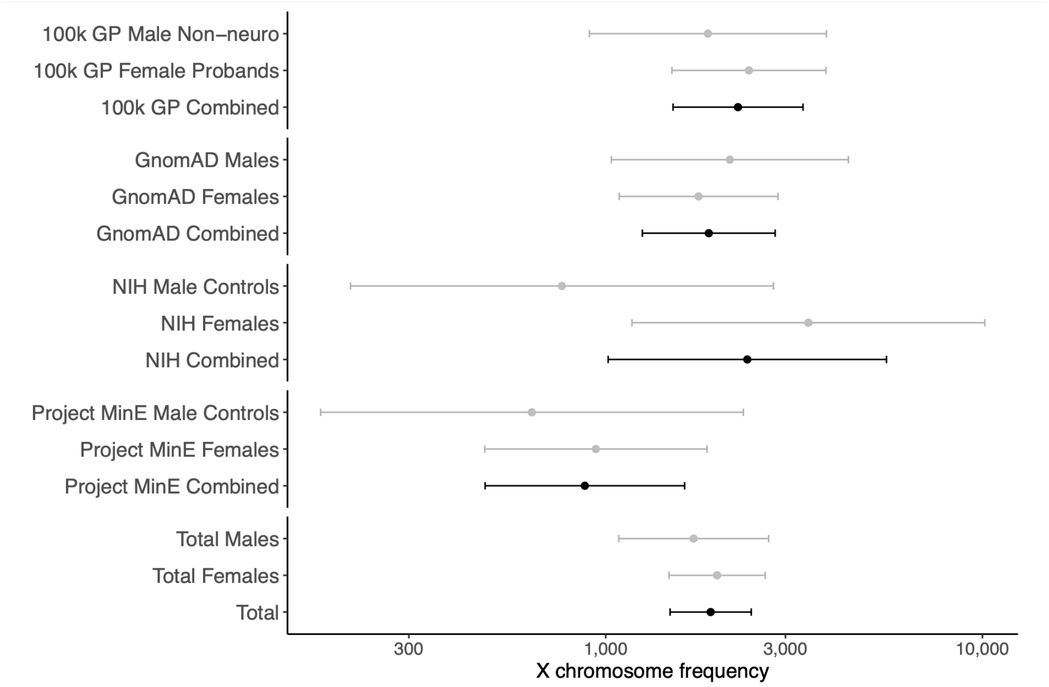

Supplementary Figure 6: Haplotype analysis. **(a)** Diagram showing variant shortlisting across the 100K GP samples from European ancestry. Brown square indicates the region of interest. **(b) (top)** Table displaying the resulting haplotypes with their occurrence in our cohort (in brackets frequencies in cases and controls); red and blue indicate significant associations for variants and haplotypes, respectively. **(bottom)** LD plot displaying the blocks resulting from the 20 variants shortlisted in (a). The region surrounding the CAG repeat is not included in any block.

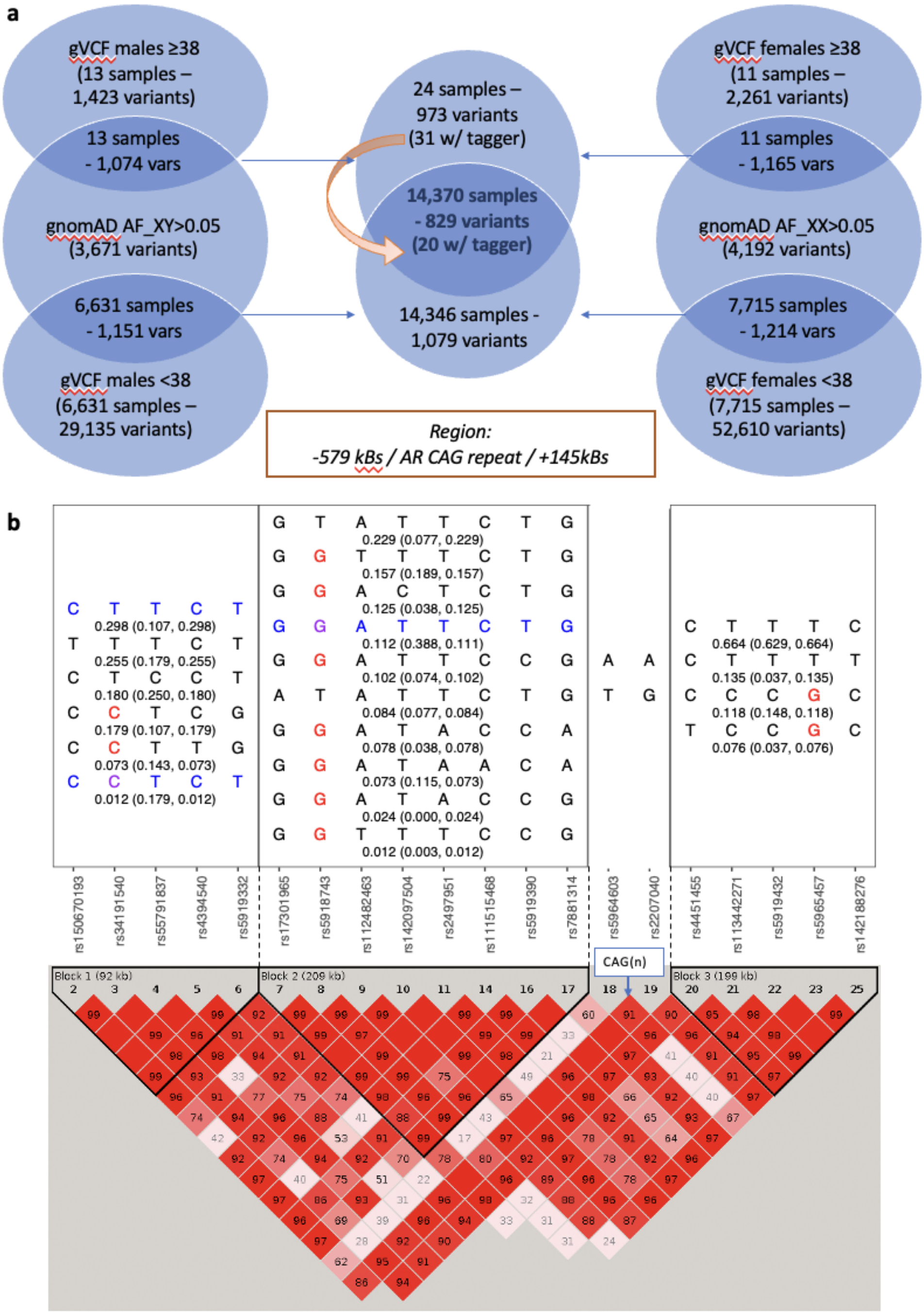

Supplement: awad050_Supplementary_Data [file awad050_supplementary_data.zip › brain-2022-01441-File006.pdf]
