# Supplementary material for: Unexpected frequency of the pathogenic AR CAG repeat expansion in the general population
Source: Brain. 2023 Feb 17;146(7):2723–9. doi: 10.1093/brain/awad050 (PMC10316764; doi:10.1093/brain/awad050)
Supplement: awad050_Supplementary_Data [file awad050_supplementary_data.zip › brain-2022-01441-File008.pdf]

Table S1. Total number of ExpansionHunter calls before and after visual QC in each cohort assessed, with threshold at 38 and 37 respectively, plus demographics

Cohort	ExpansionHunter version	Gender	Phenotype	Total participants	Total X chromosomes	Total EH calls ≥38 before visual QC	Total EH calls ≥38 after visual QC	X chromosome frequency ≥38 (95% C.I.)	Total EH calls ≥37 before visual QC	Total EH calls ≥37 after visual QC	X chromosome frequency ≥37 (95% C.I.)	Median age (1st-3rd Q)	Ethnicity
100k GP	EHv2.5	M	Non-neuro	13,072	13,072	4	2	1/6,536 (1,793-23,833)	7	7	1/1,867 (905-3,855)	47 (17-65)	AFR 1589, AFR-AMR 257, AFR-ASI 5, AFR-EUR 4, AMR 476, AMR-ASI 166, AMR-EAS 17, AMR-EUR 1566, ASI 3385, EAS 345, EUR 35828, EUR-EAS 6
		F	All	20,400	40,800	17	11	1/3,709 (2,071-6,642)	24	17	1/2,400 (1,499-3,844)		
		Combined		33,472	53,872	21	13	1/4,144 (2,422-7,090)	31	24	1/2,245 (1,509-3,340)		
GNOMAD	EHv3.2	M	All	14,947	14,947	8	5	1/2,989 (1,277-6,998)	8	7	1/2,135 (1,035-4,408)	NA	nfe: 12766, afr: 9261, oth: 395, amr: 2064, asj: 694, fin: 3188, eas: 313, sas: 203, ami: 179
		F	All	14,116	28,232	16	11	1/2,567 (1,433-4,596)	22	16	1/1,765 (1,086-2,866)		
		Combined		29,063	43,179	24	16	1/2,699 (1,661-4,384)	30	23	1/1,877 (1,251-2,817)		
NIH	EHv3.0	M	Ctrl	1,529	1,529	7	1	1/1,529 (271-8,661)	10	2	1/765 (210-2,787)	74 (63-83)	Europe: n=2286, North America: n=3233, Not known: n=1186
		F	All	5,176	10,352	4	2	1/5,176 (1,420-18,874)	10	3	1/3,451 (1,174-10,146)		
		Combined		6,705	11,881	11	3	1/3,960 (1,347-11,645)	20	5	1/2,376 (1,015-5,563)		
MINE	EHv3.1	M	Ctrl	1,272	1,272	2	2	1/636 (175-2,319)	2	2	1/636 (175-2,319)	63 (56-70)	Europe: n=2435, South Asian: n=7, West Asian: n=4, Mixed American: n=1, Not known: n=2631
		F	All	3,765	7,530	6	3	1/2,510 (854-7,380)	10	8	1/941 (477-1,857)		
		Combined		5,037	8,802	8	5	1/1,760 (752-4,121)	12	10	1/880 (478-1,620)		
**SUMMARY**		M		30,820	30,820	21	10	1/3,082 (1,674-5,674)	27	18	1/1,712 (1,083-2,707)		
		F		43,457	86,914	43	27	1/3,219 (2,213-4,683)	66	44	1/1,975 (1,472-2,651)		
		Combined		**74,277**	**117,734**	64	**37**	**1/3,182 (2,309-4,386)**	93	62	1/1,899 (1,482-2,434)		

Table S2. Sequencing data

Cohort	Chemistry	trument mo	Read length	Coverage (average)	Genome build	Aligner	EH version	Total samples
100,000 Genomes Project	Truseq PCR-free	HiSeq X	2x150bp	36x	38	Illumina Isaac	2.5.5	56595
	Truseq PCR-free	HiSeq X	2x150bp	36x	37	Illumina Isaac	2.5.5	9840
	Truseq PCR-free	HiSeq X	2x125bp	35x	37	Illumina Isaac	2.5.5	8600
Project NIH	Truseq PCR-free	HiSeq X	2x150bp	35x	38	BWA mem	3.0.1	6705
Project MinE	PCR free	HiSeq2000	100bp - paired enc	43x	37	Illumina Isaac	3.1.2	1177
	PCR free	HiSeqX	100bp - paired enc	37x	37	Illumina Isaac	3.1.2	3902
GnomAD	670 PCR-plus, 10 PCR-free, 4 Unknown	BWA mem	2x 100bp (2%)	24x	38	BWA mem	3.2.2	668
	2063 PCR-plus, 7554 PCR-free, 18762 Unknown	BWA mem	2x 150bp	24x	38	BWA mem	3.2.2	28395

**Table S3.** Validation dataset including all AR repeat size estimate by both PCR and WGS									
Each row corresponds to a sample (`validation_id`). For each sample, the gender, experimental PCR sizes for each allele, EHv255 repeat-sizes estimations for each allele, as well as the classification for EH comparing to PCR sizes can be found. All alleles compared have a value in the Visual Inspection Short/Long allele columns. TN = True Negative; FP = False Positive; TP = True Positive; FN = False negative									
validation_id	gender	exp_PCR_a1	exp_PCR_a2	EHv255_a1_avg	EHv255_a1_after_visual_QC	classification_EHv255_a1_after_visual_QC	EHv255_a2_avg	EHv255_a2_after_visual_QC	classification_EHv255_a2_after_visual_QC
NYGC_1	male	51	--	64	47	TP	--	--	--
NYGC_2	male	49	--	53	48	TP	--	--	--
NYGC_3	male	46	--	53	44	TP	--	--	--
NYGC_4	male	45	--	54	44	TP	--	--	--
NYGC_5	male	46	--	42	42	TP	--	--	--
NYGC_6	male	45	--	57	45	TP	--	--	--
NYGC_7	male	43	--	54	43	TP	--	--	--
NYGC_8	male	49	--	55	46	TP	--	--	--
NYGC_9	male	50	--	63	47	TP	--	--	--
NYGC_10	male	46	--	52	40	TP	--	--	--
NYGC_11	male	46	--	57	46	TP	--	--	--
NYGC_12	male	46	--	54	48	TP	--	--	--
NYGC_13	male	39	--	46	41	TP	--	--	--
NYGC_14	male	45	--	63	45	TP	--	--	--
NYGC_15	male	45	--	59	45	TP	--	--	--
NYGC_16	male	45	--	39	39	TP	--	--	--
NYGC_17	female	44	28	55	43	TP	28	28	TN
NYGC_18	male	48	--	57	46	TP	--	--	--
NYGC_19	female	45	21	52	43	TP	21	21	TN
NYGC_20	male	44	--	44	44	TP	--	--	--
GE_1	male	23	--	23	23	TN	--	--	--
GE_2	female	26	27	26	26	TN	27	27	TN
GE_3	male	15	--	15	15	TN	--	--	--
GE_4	male	20	--	20	20	TN	--	--	--
GE_5	male	27	--	27	27	TN	--	--	--
GE_6	male	27	--	27	27	TN	--	--	--
GE_7	male	18	--	18	18	TN	--	--	--
GE_8	male	17	--	17	17	TN	--	--	--
GE_9	male	17	--	17	17	TN	--	--	--
GE_10	female	20	25	20	20	TN	25	25	TN
GE_11	female	21	22	21	21	TN	22	22	TN
GE_12	male	21	--	21	21	TN	--	--	--
GE_13	male	18	--	18	18	TN	--	--	--
GE_14	male	19	--	19	19	TN	--	--	--
GE_15	male	20	--	20	20	TN	--	--	--
GE_16	female	19	21	19	19	TN	21	21	TN
GE_17	male	24	--	24	24	TN	--	--	--
GE_18	female	19	24	19	19	TN	24	24	TN
GE_19	male	22	--	22	22	TN	--	--	--
GE_20	female	20	23	20	20	TN	23	23	TN
GE_21	female	21	25	21	21	TN	25	25	TN
GE_22	male	21	--	21	21	TN	--	--	--
GE_23	female	17	21	17	17	TN	21	21	TN
GE_24	male	23	--	23	23	TN	--	--	--
GE_25	female	25	35	25	25	TN	35	35	TN
GE_26	male	35	--	35	35	TN	--	--	--
GE_27	male	36	--	36	36	TN	--	--	--
GE_28	male	39	--	39	39	TP	--	--	--
GE_29	male	39	--	54	40	TP	--	--	--
GE_30	female	21	36	21	21	TN	52	34	TN
GE_31	male	32	--	32	32	TN	--	--	--
GE_32	male	21	--	21	21	TN	--	--	--
GE_33	male	31	--	31	31	TN	--	--	--
GE_34	female	20	37	20	20	TN	37	37	TN
GE_35	male	24	--	24	24	TN	--	--	--
GE_36	male	21	--	21	21	TN	--	--	--
GE_37	male	35	--	35	35	TN	--	--	--

GE_38	female	18	35	18	18	TN	35	35	TN
GE_39	female	20	34	20	20	TN	34	34	TN
GE_40	female	22	42	22	22	TN	42	42	TP
GE_41	female	13	42	13	13	TN	42	42	TP
GE_42	female	29	39	29	29	TN	39	39	TP
GE_43	male	31	--	31	31	TN	--	--	--
GE_44	male	40	--	40	40	TP	--	--	--
GE_45	male	23	--	23	23	TN	--	--	--
GE_46	female	23	24	23	23	TN	52	24	TN
GE_47	female	26	35	26	26	TN	35	35	TN
GE_48	male	31	--	31	31	TN	--	--	--
GE_49	male	49	--	52	49	TP	--	--	--
GE_50	female	22	49	22	22	TN	54	47	TP
GE_51	female	18	36	18	18	TN	36	36	TN
GE_52	male	23	--	23	23	TN	--	--	--
GE_53	male	52	--	62	44	TP	--	--	--
GE_54	female	23	35	23	23	TN	52	35	TN
GE_55	male	35	--	35	35	TN	--	--	--
GE_56	female	19	37	19	19	TN	37	37	TN
GE_57	female	20	39	20	20	TN	39	39	TP
GE_58	female	14	39	14	14	TN	39	39	TP
GE_59	female	22	33	22	22	TN	34	34	TN
GE_60	male	41	--	41	41	TP	--	--	--
GE_61	male	35	--	35	35	TN	--	--	--
GE_62	male	45	--	43	43	TP	--	--	--
GE_63	male	34	--	34	34	TN	--	--	--
GE_64	female	22	34	22	22	TN	34	34	TN
GE_65	female	23	41	23	23	TN	41	41	TP
GE_66	female	26	35	26	26	TN	35	35	TN
GE_67	female	26	35	26	26	TN	35	35	TN
GE_68	male	43	--	43	43	TP	--	--	--
GE_69	female	22	23	22	22	TN	37	37	TN
GE_70	female	20	42	20	20	TN	57	42	TP
GE_71	female	22	37	22	22	TN	52	**39**	FP
GE_72	female	26	35	26	26	TN	35	35	TN
GE_73	male	21	--	21	21	TN	--	--	--
GE_74	male	21	--	21	21	TN	--	--	--
GE_75	female	18	34	18	18	TN	34	34	TN
GE_76	male	positive	--	40	40	TP	--	--	--
GE_77	male	positive	--	57	44	TP	--	--	--

Table S4. Clinical data for 100K GP samples

Age group (years)	Gender	Neurology and neurodevelopmental disorders	Ophthalmological disorders	Hearing and ear disorders	Dysmorphic and congenital abnormality syndromes	Renal and urinary tract disorders	Cardiovascular disorders	Haematological disorders	Cancer
< or = 20	Males	5	0	0	0	0	0	0	1
	Females	2	1	0	0	0	0	0	0
>20	Males	7*	1	0	0	0	0	0	0
	Females	3	0	0	0	1	1	0	3

* SBMA diagnosis confirmed with local clinician
