# Supplementary material for: Unexpected frequency of the pathogenic AR CAG repeat expansion in the general population
Source: Brain. 2023 Feb 17;146(7):2723–9. doi: 10.1093/brain/awad050 (PMC10316764; doi:10.1093/brain/awad050)
Supplement: awad050_Supplementary_Data [file awad050_supplementary_data.zip › brain-2022-01441-File009.pdf]

## The Genomics England Research Consortium

John C. Ambrose[1], Prabhu Arumugam[1], Roel Bevers[1], Marta Bleda[1], Freya Boardman-Pretty[1,2], Christopher R. Boustred[1], Helen Brittain[1], Mark J. Caulfield[1,2], Georgia C. Chan[1], Greg Elgar[1,2], Tom Fowler[1], Adam Giess[1], Angela Hamblin[1], Shirley Henderson[1,2], Tim J. P. Hubbard[1], Rob Jackson[1], Louise J. Jones[1,2], Dalia Kasperaviciute[1,2], Melis Kayikci[1], Athanasios Kousathanas[1] Lea Lahnstein[1], Sarah E. A. Leigh[1], Ivonne U. S. Leong[1], Javier F. Lopez[1], Fiona Maleady-Crowe[1], Meriel McEntagart[1], Federico Minneci[1], Loukas Moutsianas[1,2], Michael Mueller[1,2], Nirupa Murugaesu[1], Anna C.Need[1,2], Peter O’Donovan[1], Chris A.Odhams[1], Christine Patch[1,2], Mariana Buongermino Pereira[1], Daniel Perez-Gil[1], John Pullinger[1], Tahrima Rahim[1], Augusto Rendon[1], Tim Rogers[1], Kevin Savage[1], Kushmita Sawant[1], Richard H. Scott[1], Afshan Siddiq[1], Alexander Sieghart[1], Samuel C. Smith[1], Alona Sosinsky[1,2], Alexander Stuckey[1], Mélanie Tanguy[1], Ana Lisa Taylor Tavares[1], Ellen R. A. Thomas[1,2], Simon R. Thompson[1], Arianna Tucci[1,2], Matthew J. Welland[1], Eleanor Williams[1], Katarzyna Witkowska[1,2], Suzanne M.Wood[1,2]

[1] Genomics England, London, UK

[2] William Harvey Research Institute, Queen Mary University of London, London, EC1M 6BQ, UK

## Project MinE ALS Sequencing Consortium

Wouter Van Rheenen[1], Sara L. Pulit[1], Annelot M. Dekker[1], Ahmad Al Khleifat[2], William J. Brands[1], Alfredo Iacoangeli[3,4], Kevin P. Kenna[5], Ersen Kavak[6], Maarten Kooyman[7], Russell L. McLaughlin[8], Bas Middelkoop[1], Matthieu Moisse[9,10,11], Raymond D. Schellevis[1], Aleksey Shatunov[2], William Sproviero[2], Gijs H. P. Tazelaar[1], Rick A. A. Van der Spek[1], Perry T. C. Van Doormaal[1], Kristel R. Van Eijk[1], Joke Van Vugt[1], A. Nazli Basak[12], Ian P. Blair[13], Jonathan D. Glass[14,15], Orla Hardiman[16,17], Winston Hide[18,19], John E. Landers[5], Jesus S. Mora[20], Karen E. Morrison[21], Stephen Newhouse[3,4,22], Wim Robberecht[10,11,23], Christopher E. Shaw[2], Pamela J. Shaw[19], Philip Van Damme[10,11,23], Michael A. Van Es[1], Naomi R. Wray[24], Ammar Al-Chalabi[2], Leonard H. Van den Berg[1], Jan H. Veldink[1]

[1] Department of Neurology, Brain Center Rudolf Magnus, University Medical Center Utrecht, Utrecht, The Netherlands

[2] Department of Basic and Clinical Neuroscience, Maurice Wohl Clinical Neuroscience Institute, King’s College London, London, UK

[3] Department of Biostatistics and Health Informatics, Institute of Psychiatry, Psychology and Neuroscience, King's College London, London, UK

[4] NIHR Biomedical Research Centre at South London and Maudsley NHS Foundation Trust and King's College London, London, UK

[5] Department of Neurology, University of Massachusetts Medical School, Worcester, MA, USA

[6] Genomize Inc. Bogazici University, Technology Transfer Region, ETAB, Istanbul, Turkey

[7] SURFsara, Amsterdam, The Netherlands

[8] Population Genetics Laboratory, Smurfit Institute of Genetics, Trinity College Dublin, Dublin, Ireland

[9] Department of Neurosciences, KU Leuven - University of Leuven, Experimental Neurology and Leuven Research Institute for Neuroscience and Disease (LIND), B-3000 Leuven, Belgium

[10] VIB, Vesalius Research Center, Laboratory of Neurobiology, Leuven, Belgium

[11] Department of Neurology, University Hospitals Leuven, Leuven, Belgium

[12] Neurodegeneration Research Laboratory, Bogazici University, Istanbul, Turkey

[13] Centre for Motor Neuron Disease Research, Faculty of Medicine and Health Sciences, Macquarie University, Sydney, NSW 2109, Australia

[14] Department Neurology, Emory University School of Medicine, Atlanta, GA, USA

[15] Emory ALS Center, Emory University School of Medicine, Atlanta, GA, USA

[16] Academic Unit of Neurology, Trinity College Dublin, Trinity Biomedical Sciences Institute, Dublin, Ireland

[17] Department of Neurology, Beaumont Hospital, Dublin, Ireland

[18] Biostatistics Department, Harvard School of Public Health, Boston, MA, USA

[19] Sheffield Institute for Translational Neuroscience (SITraN), University of Sheffield, Sheffield, UK

[20] Department of Neurology, Hospital San Rafael, Madrid, Spain

[21] Faculty of Medicine, University of Southampton, Southampton, UK

[22] Farr Institute of Health Informatics Research, UCL Institute of Health Informatics, University College London, London, UK

[23] Department of Neurosciences, Experimental Neurology and Leuven Research Institute for Neuroscience and Disease (LIND), KU Leuven - University of Leuven, B-3000 Leuven, Belgium

[24] Queensland Brain Institute, The University of Queensland, Brisbane, Qld, Australia
