# Supplementary material for: Unexpected frequency of the pathogenic AR CAG repeat expansion in the general population
Source: Brain. 2023 Feb 17;146(7):2723–9. doi: 10.1093/brain/awad050 (PMC10316764; doi:10.1093/brain/awad050)
Supplement: awad050_Supplementary_Data [file Figure_S1.pdf › Figure_S1.pdf]

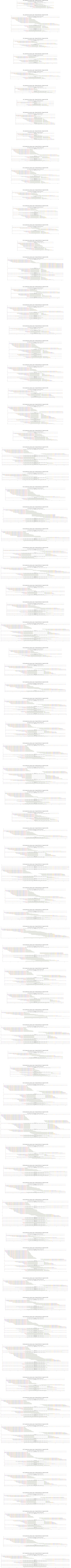

GE_1_alignments_relevant_reads - Repeat ID:AR_CAG - Repeat Unit:CAG
GE_2_alignments_relevant_reads - Repeat ID:AR_CAG - Repeat Unit:CAG
GE_3_alignments_relevant_reads - Repeat ID:AR_CAG - Repeat Unit:CAG
GE_4_alignments_relevant_reads - Repeat ID:AR_CAG - Repeat Unit:CAG
GE_5_alignments_relevant_reads - Repeat ID:AR_CAG - Repeat Unit:CAG
GE_6_alignments_relevant_reads - Repeat ID:AR_CAG - Repeat Unit:CAG
GE_7_alignments_relevant_reads - Repeat ID:AR_CAG - Repeat Unit:CAG
GE_8_alignments_relevant_reads - Repeat ID:AR_CAG - Repeat Unit:CAG
GE_9_alignments_relevant_reads - Repeat ID:AR_CAG - Repeat Unit:CAG
GE_10_alignments_relevant_reads - Repeat ID:AR_CAG - Repeat Unit:CAG
GE_11_alignments_relevant_reads - Repeat ID:AR_CAG - Repeat Unit:CAG
GE_12_alignments_relevant_reads - Repeat ID:AR_CAG - Repeat Unit:CAG
GE_13_alignments_relevant_reads - Repeat ID:AR_CAG - Repeat Unit:CAG
GE_14_alignments_relevant_reads - Repeat ID:AR_CAG - Repeat Unit:CAG
GE_15_alignments_relevant_reads - Repeat ID:AR_CAG - Repeat Unit:CAG
GE_16_alignments_relevant_reads - Repeat ID:AR_CAG - Repeat Unit:CAG
GE_17_alignments_relevant_reads - Repeat ID:AR_CAG - Repeat Unit:CAG
GE_18_alignments_relevant_reads - Repeat ID:AR_CAG - Repeat Unit:CAG
GE_19_alignments_relevant_reads - Repeat ID:AR_CAG - Repeat Unit:CAG
GE_20_alignments_relevant_reads - Repeat ID:AR_CAG - Repeat Unit:CAG
GE_21_alignments_relevant_reads - Repeat ID:AR_CAG - Repeat Unit:CAG
GE_22_alignments_relevant_reads - Repeat ID:AR_CAG - Repeat Unit:CAG
GE_23_alignments_relevant_reads - Repeat ID:AR_CAG - Repeat Unit:CAG
GE_24_alignments_relevant_reads - Repeat ID:AR_CAG - Repeat Unit:CAG
GE_25_alignments_relevant_reads - Repeat ID:AR_CAG - Repeat Unit:CAG
GE_26_alignments_relevant_reads - Repeat ID:AR_CAG - Repeat Unit:CAG
GE_27_alignments_relevant_reads - Repeat ID:AR_CAG - Repeat Unit:CAG
GE_28_alignments_relevant_reads - Repeat ID:AR_CAG - Repeat Unit:CAG
GE_29_alignments_relevant_reads - Repeat ID:AR_CAG - Repeat Unit:CAG
GE_30_alignments_relevant_reads - Repeat ID:AR_CAG - Repeat Unit:CAG
GE_31_alignments_relevant_reads - Repeat ID:AR_CAG - Repeat Unit:CAG
GE_32_alignments_relevant_reads - Repeat ID:AR_CAG - Repeat Unit:CAG
GE_33_alignments_relevant_reads - Repeat ID:AR_CAG - Repeat Unit:CAG
GE_34_alignments_relevant_reads - Repeat ID:AR_CAG - Repeat Unit:CAG
GE_35_alignments_relevant_reads - Repeat ID:AR_CAG - Repeat Unit:CAG
GE_36_alignments_relevant_reads - Repeat ID:AR_CAG - Repeat Unit:CAG
GE_37_alignments_relevant_reads - Repeat ID:AR_CAG - Repeat Unit:CAG
GE_38_alignments_relevant_reads - Repeat ID:AR_CAG - Repeat Unit:CAG
GE_39_alignments_relevant_reads - Repeat ID:AR_CAG - Repeat Unit:CAG
GE_40_alignments_relevant_reads - Repeat ID:AR_CAG - Repeat Unit:CAG
GE_41_alignments_relevant_reads - Repeat ID:AR_CAG - Repeat Unit:CAG
GE_42_alignments_relevant_reads - Repeat ID:AR_CAG - Repeat Unit:CAG
GE_43_alignments_relevant_reads - Repeat ID:AR_CAG - Repeat Unit:CAG
GE_44_alignments_relevant_reads - Repeat ID:AR_CAG - Repeat Unit:CAG
GE_45_alignments_relevant_reads - Repeat ID:AR_CAG - Repeat Unit:CAG
GE_46_alignments_relevant_reads - Repeat ID:AR_CAG - Repeat Unit:CAG
GE_47_alignments_relevant_reads - Repeat ID:AR_CAG - Repeat Unit:CAG
GE_48_alignments_relevant_reads - Repeat ID:AR_CAG - Repeat Unit:CAG
GE_49_alignments_relevant_reads - Repeat ID:AR_CAG - Repeat Unit:CAG
GE_50_alignments_relevant_reads - Repeat ID:AR_CAG - Repeat Unit:CAG
GE_51_alignments_relevant_reads - Repeat ID:AR_CAG - Repeat Unit:CAG
GE_52_alignments_relevant_reads - Repeat ID:AR_CAG - Repeat Unit:CAG
GE_53_alignments_relevant_reads - Repeat ID:AR_CAG - Repeat Unit:CAG
GE_54_alignments_relevant_reads - Repeat ID:AR_CAG - Repeat Unit:CAG
GE_55_alignments_relevant_reads - Repeat ID:AR_CAG - Repeat Unit:CAG
GE_56_alignments_relevant_reads - Repeat ID:AR_CAG - Repeat Unit:CAG
GE_57_alignments_relevant_reads - Repeat ID:AR_CAG - Repeat Unit:CAG
GE_58_alignments_relevant_reads - Repeat ID:AR_CAG - Repeat Unit:CAG
GE_59_alignments_relevant_reads - Repeat ID:AR_CAG - Repeat Unit:CAG
GE_60_alignments_relevant_reads - Repeat ID:AR_CAG - Repeat Unit:CAG
GE_61_alignments_relevant_reads - Repeat ID:AR_CAG - Repeat Unit:CAG
GE_62_alignments_relevant_reads - Repeat ID:AR_CAG - Repeat Unit:CAG
GE_63_alignments_relevant_reads - Repeat ID:AR_CAG - Repeat Unit:CAG
GE_64_alignments_relevant_reads - Repeat ID:AR_CAG - Repeat Unit:CAG
GE_65_alignments_relevant_reads - Repeat ID:AR_CAG - Repeat Unit:CAG
GE_66_alignments_relevant_reads - Repeat ID:AR_CAG - Repeat Unit:CAG
GE_67_alignments_relevant_reads - Repeat ID:AR_CAG - Repeat Unit:CAG
GE_68_alignments_relevant_reads - Repeat ID:AR_CAG - Repeat Unit:CAG
GE_69_alignments_relevant_reads - Repeat ID:AR_CAG - Repeat Unit:CAG
GE_70_alignments_relevant_reads - Repeat ID:AR_CAG - Repeat Unit:CAG
GE_71_alignments_relevant_reads - Repeat ID:AR_CAG - Repeat Unit:CAG
GE_72_alignments_relevant_reads - Repeat ID:AR_CAG - Repeat Unit:CAG
GE_73_alignments_relevant_reads - Repeat ID:AR_CAG - Repeat Unit:CAG
GE_74_alignments_relevant_reads - Repeat ID:AR_CAG - Repeat Unit:CAG
GE_75_alignments_relevant_reads - Repeat ID:AR_CAG - Repeat Unit:CAG
GE_76_alignments_relevant_reads - Repeat ID:AR_CAG - Repeat Unit:CAG
GE_77_alignments_relevant_reads - Repeat ID:AR_CAG - Repeat Unit:CAG
